# New Drugs, Appliances, and Things Medical

**Published:** 1892-10-08

**Authors:** 


### NEW DRUGS, APPLIANCES, AND THINGS MEDICAL.

[All preparations, appliances, novelties, etc., of which a notice is desired, should be sent for the Editor, to care of The Manager, 140, Strand, London, W.C.

### MESSRS. FRY AND SONS' COCOA AND CHOCOLATE.

In noticing Fry's Cocoa and Chocolate, we are but re-introducing a very old and tried friend. Nevertheless, this old friend is constantly putting on new garments and improving old ones. Messrs. Fry and Sons are manufacturers who do not stand still. They have realised that the public relies more and more upon its cocoa as an important means of nourishment. People will for the most part have nothing to say to the thick starchy compounds of earlier days, and demand the pure slightly bitter flavour of the cocoa nib e'er they will be satisfied. Messrs. Fry have determined to reach perfection if possible, and in their "Pure Concentrated Cocoa" as a perfectly pure and nourishing beverage they may fairly be said to have done, so. Let the sceptical pay a visit to the vast manufacturing colony at Bristol, and see for themselves the care and thoroughness of the method by which this cocoa is produced. The careful selection of the bean, the perfect roasting, the winnowing, crushing and pressing to remove superfluous fat, that is carried out, will make the observer regard this preparation not only with favour but confidence and respect. Those who are not acquainted with the delicious flavour of the cocoa we recommend to try it, and also the "Malted Cocoa," which will meet with even greater popularity in some quarters. The "malt" does not assert any foreign flavour to the cocoa, but seems if anything to accentuate that of the cocoa. Messrs. Fry's chocolates are also excellent, especially the "Caracas" and "Ceylon" descriptions, and if there are foreign firms who still hold the palm above us in the preparation of this commodity, Messrs. Fry are so little behind in the race, that we doubt not the day is not far off when this can no longer be said. By design and variety Messrs. Fry have already succeeded in making their chocolate and other bonbons most attractive, and parents may give these to their children with every assurance of their purity. The manufactory is very self-contained, the pretty boxes employed to receive their fancy productions all being made on the premises. It is pleasant to know in these days of "sweating" that Messrs. Fry have due regard to their army of workers, a fact that is in itself a recommendation to the use of their productions.

### TOILET SPECIALITIES.

We have received samples of their specialities from the Royal Perfumery Company in Bond Street, which are of an excellent description. Messrs. Price, the manufacturers, have paid especial attention to the preparation of soap, with the result that we can unhesitatingly say that some of their productions are the most agreeable we have met with. This is especially the case in regard to the Cucumber and Glycerine and Velvine Soaps. The soaps are trebly milled and dessicated, and being thus deprived of all moisture, they are admirably firm and economical in use. For those who prefer the use of some form of antiseptic, we would recommend the Eucalyptus and Coal Tar Soaps, which are most effective preparations. We have dwelt especially on the merits of Messrs. Price's soaps because we feel that in this branch of toilet specialities they have signal success, but we have also found their perfumes and other commodities of very good quality.

